# How is postoperative pain after hip and knee replacement managed? An analysis of two large hospitals in Australia

**DOI:** 10.1186/s13741-024-00403-w

**Published:** 2024-05-31

**Authors:** Giovanni E. Ferreira, Asad E. Patanwala, Hannah Turton, Aili V. Langford, Ian A. Harris, Chris G. Maher, Andrew J. McLachlan, Paul Glare, Chung-Wei Christine Lin

**Affiliations:** 1 Sydney Musculoskeletal Health, Faculty of Medicine and Health, The University of Sydney, Sydney, Australia; 2 Institute for Musculoskeletal Health, Sydney Local Health District, Sydney, Australia; 3 Pharmacy Department, Royal Prince Alfred Hospital, Sydney, Australia; 4 Centre for Medicine Use and Safety, Faculty of Pharmacy and Pharmaceutical Sciences, Monash University, Melbourne, Australia; 5 Whitlam Orthopaedic Research Centre, Ingham Institute for Applied Medical Research, Sydney, Australia; 6 Orthopaedic Department, South Western Sydney Local Health District, Liverpool Hospital, Sydney, Australia; 7 Sydney Pharmacy School, Faculty of Medicine and Health, The University of Sydney, Sydney, Australia; 8 Northern Clinical School, Faculty of Medicine & Health, University of Sydney, Sydney, Australia; 9 Royal Prince Alfred Hospital, Level 10 North, King George V Building, Missenden Road, PO Box M179, Camperdown, NSW 2050 Australia

**Keywords:** Osteoarthritis, Joint replacement, Opioids

## Abstract

**Background:**

Multimodal analgesia regimens are recommended for the postoperative period after hip and knee replacement surgeries. However, there are no data on practice patterns for analgesic use in the immediate postoperative period after hip and knee replacements in Australia.

**Objectives:**

To describe analgesic prescribing patterns in the inpatient postoperative phase for patients undergoing hip and knee replacement.

**Methods:**

Retrospective study of electronic medical record data from two major hospitals in Sydney, Australia. We identified analgesic medication prescriptions for all patients aged 18 years and older who underwent hip or knee replacement surgery in 2019. We extracted data on pain medications prescribed while in the ward up until discharge. These were grouped into distinct categories based on the Anatomical Therapeutic Chemical classification. We described the frequency (%) of pain medications used by category and computed the average oral morphine equivalent daily dose (OMEDD) during hospitalisation.

**Results:**

We identified 1282 surgeries in 1225 patients. Patients had a mean (SD) age of 69 (11.8) years; most (57.1%) were female. Over 99% of patients were prescribed opioid analgesics and paracetamol during their hospital stay. Most patients (61.4%) were managed with paracetamol and opioids only. The most common prescribed opioid was oxycodone (87.3% of patients). Only 19% of patients were prescribed nonsteroidal anti-inflammatories (NSAIDs). The median (IQR) average daily OMEDD was 50.2 mg (30.3–77.9).

**Conclusion:**

We identified high use of opioids analgesics as the main strategies for pain control after hip and knee replacement in hospital. Other analgesics were much less frequently used, such as NSAIDs, and always in combination with opioids and paracetamol.

**Supplementary Information:**

The online version contains supplementary material available at 10.1186/s13741-024-00403-w.

## Background

Hip and knee replacement are very common surgeries. The use of hip and knee replacement surgeries has been increasing rapid in Australia for many years (Ackerman et al. 2019). In 2022, there were 52,863 and 64,846 hip and knee replacement surgeries performed in Australia (Association AO 2023), making these 2 of the most common surgical procedures in the country.

While both surgeries result in large improvements in pain, function, overall health, and satisfaction (Heath et al. 2021), they are associated with significant postoperative pain (Chan et al. 2013). As poorly controlled pain in the immediate postoperative period predicts the development of chronic pain (Glare et al. 2019), guidelines currently recommend multimodal analgesia to optimise pain control (Chou et al. 2016). Multimodal analgesia can include systemic (e.g. opioid analgesics, nonsteroidal anti-inflammatories [NSAIDs]), local (e.g. local anaesthetic infiltrations), or regional pharmacological therapies (e.g. nerve blocks), as well as non-pharmacological therapies (e.g. electrical stimulation). The exact components of effective multimodal analgesia are unknown and likely vary according to patients, surgical procedure, and setting (Chou et al. 2016).

The evidence for different pain medications used in multimodal pain management regimens is variable. There is some evidence from a small number of trials that NSAIDs provide small to moderate reductions in postoperative pain and opioid use compared to placebo (Fillingham et al. 2020). In contrast, paracetamol is not effective to reduce postoperative pain following hip and knee replacement (Abdel Shaheed et al. 2021). Less commonly used pain medications such as gabapentinoids have been shown to provide small reductions in postoperative pain and opioid use after surgery (including orthopaedic surgery) (Verret et al. 2020); however, perioperative use of gabapentin has been associated with increased harms such as delirium and pulmonary complications (Park et al. 2022; Ohnuma et al. 2020). Anaesthetics such as ketamine were shown to be effective at reducing postoperative pain (but not amongst patients undergoing joint replacement surgery) and opioid use (Riddell et al. 2019). Systemic steroids have also been shown to provide small reductions in pain and opioid use compared to placebo after total knee replacement (Gasbjerg et al. 2022; Lunn and Kehlet 2013). Despite the variable evidence of these drugs on postsurgical pain, multimodal pain regimens have been associated with reduced length of stay and opioid use (Memtsoudis et al. 2018).

Multimodal pain management is common amongst patients undergoing hip and knee replacement. An American population-based observational study found that 85.6% of patients undergoing hip and knee replacements received multimodal pain management, defined as the use of at least one type of opioid and at least one more non-opioid pain medication (Memtsoudis et al. 2018). The most commonly used non-opioid pain medications reported in that study were NSAIDs and paracetamol, followed by peripheral nerve blocks, gabapentinoids (e.g. pregabalin, gabapentin), ketamine, and corticosteroids.

Australia is amongst the countries with the highest rates of knee and hip replacements in the world (OECD 2019). However, to the best of our knowledge, there are no data on practice patterns for analgesic use in the immediate postoperative period after hip and knee replacements in Australia. The aims of this study are to describe analgesic prescribing patterns in the inpatient postoperative phase for patients undergoing hip and knee replacement.

## Methods

### Participants and setting

This is a retrospective study of routinely collected electronic medical record data reported per the RECORD guidelines (Benchimol et al. 2015). This study was approved by the Sydney Local Health District (Royal Prince Alfred Zone) Ethics Committee (approval number: X22-0047 & 2022/ETH00330).

We identified pain medication prescriptions of all patients aged 18 years and older who underwent hip and knee replacement in the 2019 calendar year in two major teaching public hospitals in Sydney, Australia: Concord Repatriation General Hospital and Royal Prince Alfred Hospital. These hospitals serve more than 700,000 people living within their catchment area. We chose 2019 as it was the last year before the Covid-19 pandemic, and thus, there were no restrictions or limitations in the number of elective surgeries performed at those hospitals. Hip and knee replacements included elective, trauma, and revision surgeries.

Patients were identified using the Australian Refined Diagnosis-Related Groups (AR-DRGs) version 9.0. AR-DRG provides a clinically meaningful way to relate the number and type of patients treated in a hospital to the resources required by the hospital (Australian Institute of Health and Welfare 2022). AR-DRGs group patients with similar diagnoses requiring similar hospital services. Episodes of admitted acute care are assigned with disease and intervention codes by health information managers or clinical coders. AR-DRGs are then assigned based on these codes. The AR-DRG codes classify surgeries according to complexity (major/minor), primary versus revision surgery, or trauma status (non-trauma/trauma). AR-DRG codes used in this study are described in Appendix 1. Complexity refers to a measure that quantifies relative levels of resource utilisation within each diagnostic group and is used to split diagnostic groups into different DRG levels on the basis of resource homogeneity (Independent Health and Aged Care Pricing Authority 2019).

### Data sources and collection

We queried patients’ electronic medical records and exported data into a web-based standardised data collection form in Research Electronic Data Capture (REDCap) (Harris et al. 2019). We collected patient data (e.g. age, sex), admission data (e.g. hospital length of stay), and analgesic prescriptions. We determined, a priori, a list of medications that are commonly used for pain management and grouped them into distinct categories based on the Anatomical Therapeutic Chemical (ATC) classification (Who Collaborating Centre for Drug Statistics Methodology 2021). The list of analgesics is described in Appendix 2. We grouped analgesics into the following categories: paracetamol, nonsteroidal anti-inflammatories (NSAIDs), opioids, muscle relaxants, antiepileptics, antidepressants, and anaesthetics.

We captured analgesic prescriptions from when the patient arrived at the ward post-surgery until hospital discharge. We did not capture analgesics prescribed intraoperatively or in the ICU (when ICU stay was required). We had planned to obtain information on local and regional anaesthetics (e.g. femoral nerve blocks which ropivacaine which is commonly used) and patient-controlled analgesia; however, this information was not available as medications used in these analgesic modalities are not charted in the electronic medical record. The codes used to extract information from the patients’ electronic medical records was reviewed by another researcher who is an experienced hospital pharmacist, thus ensuring the accuracy of our data.

Prescribed analgesic prescriptions contained information on the type of pain medicine, the dose, frequency of use per day, the administration route (e.g. oral), whether the prescription was scheduled or as needed (PRN), and duration of therapy. For prescriptions where a dose range was provided (e.g. ‘oxycodone 5–10 mg’), we assumed that the lowest dose was administered. When the duration of a prescription was zero, we assumed that the analgesic was administered once based on our knowledge of how data is captured in the electronic system. We assumed that the dose of oxycodone-naloxone was 5 mg/2.5 mg, and that the dose of tramadol was 50 mg, as these were the only opioid analgesics with missing information regarding dose regimens.

### Outcomes and analyses

We described the frequency and proportion of analgesics used. We presented this information by ATC category (e.g. opioids, N02A) and by drug (e.g. oxycodone) for the overall sample and stratified by type of surgery (hip vs knee replacement).

We computed the average oral morphine equivalent daily dose (OMEDD) by dividing the cumulative opioid OMEDD during hospitalisation by the length of stay (in days). The cumulative opioid OMEDD was calculated by summing the opioid OMEDD for all opioid prescriptions during the patient’s hospital stay. For each opioid prescription, the OMEDD was calculated by multiplying the dose of the opioid by the number of times the opioid was to be given to the patient per day by the duration of the prescription (in hours) and by the opioid-specific conversion factor using the Australian and New Zealand College of Anaesthetists Opioid Dose Equivalence Calculation Table (Appendix 3) (ANZCA 2021). We assumed that *pro re nata* (PRN) prescriptions with frequencies less than every 4 h were administered a maximum of six times in a day. The base assumption was that a PRN analgesic would be administered 50% of the time that it was eligible for administration. For example, a PRN analgesic prescription that was indicated for administration every 4 h (i.e. six times in a given day) was assumed to be administered to a patient three times in a given day (i.e. six eligible administrations per day divided by 2). This is based on the experience of the investigators (Stasinopoulos et al. 2018). However, this assumption was varied from 25 to 100% in a sensitivity analysis. We reported OMEDD data for the overall sample and for specific subgroups: type of surgery, complexity as determined by the AR-DRG code assigned to the patient (e.g. revision status [primary versus revision], trauma status, and whether patients received non-opioid pain medications other than paracetamol). Pain medication other than opioids and paracetamol refers to NSAIDs, antiepileptics, antidepressants, anaesthetics, and corticosteroids. We used one-way ANOVA tests to compare the OMEDD between categories of each specific subgroup. One-way ANOVA was used because it is robust to violations of normality as was the case with our OMEDD data (Blanca et al. 2017). We considered *p*-values < 0.05 as statistically significant. All analyses were conducted in Stata 17 (College Station, TX, USA).

## Results

### Characteristics of patients

We identified 1282 surgeries in 1225 patients in the two hospitals. Patients had a mean (SD) age of 69 (11.8) years, and most (57.1%) were female. Most surgeries were elective (92.5%), and 724 (56.4%) were knee replacements (Table 1). All knee replacements were elective. The overall median (IQR) length of stay was 5 (3–7) days. The median (IQR) length of stay was shorter for primary (4 days [3–6]) in relation to revision surgeries (8 days [5–16]).

**Table 1:** Patient and surgery characteristics

	Total
Patient characteristics (*n* = 1225 patients)	
Age, mean (SD)	69 (11.8)
Age (≥ 65 years), *n* (%)	872 (68.1)
Sex, *n* (%)	
Female	732 (57.1)
Male	550 (42.9)
Surgery characteristics (*n* = 1282 surgeries)	
Type of surgery, *n* (%)	
Hip replacement	558 (43.5)
Knee replacement	724 (56.4)
Elective surgery, *n* (%)	1186 (92.5)
Major complexity, *n* (%)	210 (16.4)
Revision, *n* (%)	111 (8.7)
Length of stay (days), median (IQR)	5 (3–7)

### Analgesic prescriptions

A total of 7696 analgesic prescriptions were prescribed in the postoperative period during hospitalisation. All patients had at least one analgesic prescription; the median number of prescriptions per patient was 5 (4–7) over the duration of their hospital stay (Appendices 4 and 5).

Over 99% of patients received an opioid analgesic and paracetamol during their hospital stay. Only one patient (0.1%) did not receive an opioid analgesic during their hospital stay. Most patients (61.4%) were managed with paracetamol and opioids only. The most common prescribed opioid analgesic was oxycodone (87.3% of all opioid prescriptions), followed by tapentadol (62.5%), and fentanyl (26.1%). Other analgesics were prescribed much less often. Only 19% of patients had at least one prescription for NSAIDs, 12.1% for antiepileptics, 9.1% for corticosteroids, 8.2% for antidepressants, 1.3% for anaesthetics (e.g. ketamine), and 0.2% for muscle relaxants (Table 2). The type and frequency of analgesics prescribed were similar between patients undergoing primary versus revision surgery (Appendix 6).

**Table 2:** Frequency (%) of surgeries that had at least one prescription for each medication and drug class

	Hip replacement (*n* = 558)	Knee replacement (*n* = 724)	Total (*n* = 1282)
Opioids	557 (99.8)	724 (100)	1281 (99.9)
Oxycodone	488 (87.5)	631 (87.2)	1119 (87.3)
Tapentadol	322 (57.7)	479 (66.2)	801 (62.5)
Fentanyl	152 (27.2)	182 (25.1)	334 (26.1)
Morphine	166 (29.8)	167 (23.1)	333 (26)
Buprenorphine	122 (21.9)	210 (29)	332 (25.9)
Tramadol	83 (14.9)	68 (9.4)	151 (11.8)
Oxycodone-naloxone	74 (13.3)	52 (7.2)	126 (9.8)
Hydromorphone	25 (4.5)	13 (1.8)	38 (3)
Codeine	4 (0.7)	0	4 (0.3)
Paracetamol	556 (99.6)	723 (99.9)	1279 (99.8)
Paracetamol	556 (99.6)	723 (99.9)	1279 (99.8)
Nonsteroidal anti-inflammatories	106 (19)	138 (19.1)	244 (19)
Aspirin (dose ≥ 150 mg/day)	37 (6.6)	67 (9.3)	104 (8.1)
Celecoxib	37 (6.6)	46 (6.4)	83 (6.5)
Ibuprofen	12 (2.2)	14 (1.9)	26 (2)
Diclofenac	9 (1.6)	7 (1)	16 (1.3)
Indomethacin	5 (0.9)	2 (0.3)	7 (0.6)
Parecoxib	4 (0.7)	3 (0.4)	7 (0.6)
Naproxen	4 (0.7)	2 (0.3)	6 (0.5)
Ketoprofen	2 (0.4)	0	2 (0.2)
Ketorolac	1 (0.2)	1 (0.1)	2 (0.2)
Piroxicam	0	1 (0.1)	1 (0.1)
Antiepileptics	64 (11.5)	91 (12.6)	155 (12.1)
Pregabalin	53 (9.5)	80 (11.1)	133 (10.4)
Gabapentin	8 (1.4)	8 (1.1)	16 (1.3)
Carbamazepine	3 (0.5)	5 (0.7)	8 (0.6)
Topiramate	0	2 (0.3)	2 (0.2)
Corticosteroids	65 (11.7)	52 (7.2)	117 (9.1)
Dexamethasone	39 (7)	30 (4.1)	69 (5.4)
Prednisolone	24 (4.3)	20 (2.8)	44 (3.4)
Hydrocortisone	6 (1.1)	6 (0.8)	12 (0.9)
Methylprednisolone	1 (0.2)	0	1 (0.1)
Antidepressants	40 (7.2)	65 (9)	105 (8.2)
Amitriptyline	27 (4.8)	30 (4.1)	57 (4.5)
Venlafaxine	8 (1.4)	20 (2.8)	28 (2.2)
Duloxetine	7 (1.3)	15 (2.1)	22 (1.7)
Anaesthetics	7 (1.3)	10 (1.4)	17 (1.3)
Ketamine	7 (1.3)	10 (1.4)	17 (1.3)
Muscle relaxants	2 (0.4)	0	2 (0.2)
Baclofen	2 (0.4)	0	2 (0.2)

Values may not add up to 100% due to rounding

### Oral morphine equivalent daily dose (OMEDD)

The overall median (IQR) average daily OMEDD was 50.2 (30.3 to 77.9) mg for the whole sample. Type of surgery (hip or knee replacement), whether the surgery was classified as major or minor complexity, and whether it was a primary or revision surgery did not have an effect on OMEDD (Table 3). Patients who underwent either hip or knee replacement due to trauma had lower OMEDD compared to those who had elective surgery (median OMEDD: 30.7 vs 52.2; *p* < 0.001). Similarly, patients who were prescribed another pain medicine in addition to paracetamol and opioid analgesics had higher OMEDD compared to those who only received opioid analgesics and paracetamol (median OMEDD: 62.2 vs 46; *p* < 0.001) (Table 3). Results were similar in the sensitivity analysis that varied the extent of analgesic use in PRN prescriptions (Appendix 7).

**Table 3:** Median (IQR) oral morphine equivalent daily dose (OMEDD) for the total sample and key subgroups of patients

	*N*	OMEDD	*p*-value
Total sample	1282	50.2 (30.3–77.9)	
Type of surgery			
Hip replacement	558	48 (27.6–80.6)	0.51
Knee replacement	724	52.5 (32.1–76.9)	
Trauma status			
Yes	1186	52.2 (31.7–79.4)	< 0.001
No (elective surgery)	96	30.7 (18.9–48.2)	
Complexity			
Major	210	46.8 (25.6–73)	0.34
Minor	1072	51.1 (30.9–79)	
Revision surgery			
Yes	111	49.1 (30.2–73.4)	0.77
No	1171	50.5 (30.3–78.8)	
Pain medicines received			
Opioids and paracetamol only	787	46 (26.3–68.3)	< 0.001
Opioids, paracetamol, and other pain medicines	495	62.2 (36.1–90)	

## Discussion

We described the use of analgesics for postoperative pain management in hospitalised patients undergoing hip and knee replacement surgeries at two major hospitals in Sydney, Australia. We found 99% of patients were prescribed opioid analgesics and paracetamol during their hospital stay. Other non-opioid pain medicines were much less frequently prescribed. For example, NSAIDs were the second most commonly prescribed non-opioid analgesic, but only 19% of patients in our study received them during their hospital stay. We found no differences in OMEDD depending on type of surgery (hip or knee replacement), the complexity of surgery (major or minor), or whether it was a primary or revision surgery. Patients undergoing trauma surgery had lower OMEDD than those undergoing elective surgery, and those who were prescribed other pain medicines in addition to opioid analgesics and paracetamol had higher OMEDD compared to those who were only prescribed opioid analgesics and paracetamol.

To the best of our knowledge, this is the first study describing analgesic prescribing patterns in patients who have undergone hip and knee replacement are treated for pain postoperatively while in hospital in Australia. Our findings demonstrate that opioids are used as first-line analgesics postoperatively, as over 99% of patients in our study had at least one opioid prescription during their hospital stay.

Oxycodone was the most commonly opioid analgesic prescribed to patients in our study. This finding reflects opioid analgesic prescribing practices in Australia, where oxycodone is the most commonly prescribed prescription opioid. In 2016–2017, there were 5.7 million prescriptions prescribed to 1.3 million people (Australian Institute of Health and Welfare. 2018). Oxycodone is associated with adverse effects including constipation, sedation, delirium, and respiratory depression (Liu et al. 2024).

Tapentadol was another commonly prescribed opioid in our study, in line with findings from a recent Australian study that showed a 223% increase in hospital prescriptions for tapentadol, making it the most prescribed opioid in the hospitals included in that study (Mirabella et al. 2022). Tapentadol, which was first approved in Australia in 2011, is a new synthetic opioid considered to have a dual mechanism of action that targets both nociceptive and neuropathic components of postoperative pain. Higher doses of tapentadol (e.g. 75 or 100 mg) have similar efficacy compared to oxycodone for pain and result in fewer gastrointestinal adverse events such as nausea and constipation (Wang et al. 2020). A recent Australian study has found that, compared to oxycodone, tapentadol may be more cost-effective for the treatment of postoperative pain after major hip surgeries (hip replacement and other surgeries for hip fractures) (Wang et al. 2022a). However, there are concerns about the burden of out-of-pocket cost to patients who may be discharged from hospital with a prescription of tapentadol since the immediate release formulation is currently not subsided by the Australian government under the Pharmaceutical Benefits Schedule (Mirabella et al. 2022).

The use of non-opioid analgesics postoperatively plays a key role in reducing opioid requirements and associated adverse events. While some studies have reported an association between more use of non-opioid analgesics and reduction in opioid consumption (Memtsoudis et al. 2018), we found the opposite. Compared to patients who only had opioid analgesics and paracetamol prescribed, patients receiving at least one prescription for NSAIDs, antiepileptics, antidepressants, anaesthetics, or corticosteroids received, on average, 16.2 OMEDD more. These findings may indicate that non-opioid analgesics are being used in those who undergo more complex surgeries and who may not have achieved satisfactory pain control with opioids and paracetamol only rather than as a first-line strategy to reduce the need for opioid medicines. Interestingly, we also found that patients undergoing surgery due to trauma had lower OMEDD than those undergoing elective surgery. One explanation for these findings is that there could be a higher proportion of patients amongst those undergoing elective surgery who were already using opioid analgesics prior to surgery, which is a known independent risk factors for increased postoperative opioid analgesia requirement (Rozell et al. 2017). We did not find differences in opioid prescription between those undergoing hip and knee replacement, as opposed to previous studies that found higher opioid use amongst patients undergoing knee compared to hip replacement (Roebke et al. 2020).

Recent evidence has called into question the effectiveness of opioid analgesics to manage postsurgical pain compared to non-opioid analgesics. A recent trial has shown that in patients undergoing knee or shoulder arthroscopy, an open-label multimodal opioid-sparing protocol significantly reduced inpatient opioid use without having any effect on patient outcomes (e.g. pain) (No Pain Investigators 2022). Another recent trial, in patients with fractures managed surgically, treatment with oxycodone at discharge was not superior to codeine and paracetamol combined on reducing pain and improving quality of life (Jenkin et al. 2021). Recent evidence has also shown that use of serotonin-norepinephrine reuptake inhibitor antidepressants significantly reduced postoperative pain compared to placebo, as well as opioid in the postoperative phase (Ferreira et al. 2023; Wang et al. 2022b). Whether this approach would be equally effective after major surgery such as joint replacements is unclear and needs to be investigated.

On average, patients in our study received lower doses of opioid (median OMEDD 50.2), which contrasts with findings from previous studies. For example, an American study by Memtsoudis et al. found that in patients who underwent hip and knee replacements, daily OMEDD ranged from 300 (in those who received less than 2 types of analgesics) to 205 (in those who received more than 2 types of analgesics) (Memtsoudis et al. 2018).

Our study has limitations. We were unable to include data for local or regional anaesthetics and patient-controlled analgesia, which are routinely used for the management of pain in the two hospitals included in this study. The implications are that the OMEDD values presented in this study are likely to represent an underestimation of the amount of opioids that patient consumed daily while in hospital. We made assumptions about some of the prescriptions, such as those where a dose or duration of the prescription was not clear. The OMEDD is an estimate based on prescription rather than documented administrations. Whether these assumptions over- or underestimated opioid consumption is unknown. Validation of these assumptions by future studies would be important. This study also has several strengths. We provided a detailed breakdown of types of opioid and non-opioid analgesics used. Previous studies have only described analgesics used in the postoperative period using broad category descriptors. Our data captured all patients who underwent hip and knee replacement at the two participating hospitals, therefore providing a representative picture of standard of care. We had no access to the rationale for each medication prescription or other clinical variables that could have helped investigate our hypothesis. Future work should focus on identifying whether multimodal analgesia regimens are used as part of standard of care or only in those who failed an initial course of opioids and paracetamol. We did not capture analgesics prescribed intraoperatively or in the ICU (when ICU stay was required) and did not collect patient-reported outcomes such as pain, patient comorbidities, or preoperative opioid use, which could have helped explain the observed prescribing patterns in our sample.

## Conclusions

We identified high use of opioids and paracetamol as the main strategies for pain control after hip and knee surgery in hospitalised patients. Other pain medicines, notably NSAIDs, were much less frequently used and always in combination with opioids and paracetamol.

### Supplementary Information

Supplementary Material 1: Appendix 1. Eligible AR-DRG codes. Hip surgery. Knee surgery. Appendix 2. List of pain medicines that we searched for. Appendix 3. Opioid Dose Equivalence Calculation Table. Appendix 4. Distribution of total number of prescriptions. Appendix 5. Number of prescriptions per pain medication class (*n* = 7696 orders). Appendix 6. Frequency (%) of surgeries that had at least one prescription for each medication and drug class stratified by revision status (primary versus revision). Appendix 7. Sensitivity analysis considering that 25% (OMEDD 25%), 75% (OMEDD 75%), and 100% (OMED 100%) of PRN opioid orders were prescribed to patients.

## Data Availability

The data that support the findings of this study are available from Sydney Local Health District. Restrictions apply to the availability of these data, which were used under license for this study. Data are available from the authors with the permission of Sydney Local Health District Ethics Committee.

## Abbreviations

NSAIDs: Nonsteroidal anti-inflammatories
AR-DRG: Australian Refined Diagnosis-Related Groups
OMEDD: Oral morphine equivalent daily dose
PRN: Pro re nata
SD: Standard deviation
IQR: Interquartile range
