# Supplementary material for: How is postoperative pain after hip and knee replacement managed? An analysis of two large hospitals in Australia
Source: Perioper Med (Lond). 2024 May 31;13:49. doi: 10.1186/s13741-024-00403-w (PMC11143609; doi:10.1186/s13741-024-00403-w)
Supplement: Supplementary file 1 — Supplementary Material 1: Appendix 1. Eligible AR-DRG codes. Hip surgery. Knee surgery. Appendix 2. List of pain medicines that we searched for. Appendix 3. Opioid Dose Equivalence Calculation Table. Appendix 4. Distribution of total number of prescriptions. Appendix 5. Number of prescriptions per pain medication class (n = 7696 orders). Appendix 6. Frequency (%) of surgeries that had at least one prescription for each medication and drug class stratified by revision status (primary versus revision). Appendix 7. Sensitivity analysis considering that 25% (OMEDD 25%), 75% (OMEDD 75%), and 100% (OMED 100%) of PRN opioid orders were prescribed to patients. [file 13741_2024_403_MOESM1_ESM.docx]

**Appendix 1. Eligible AR-DRG codes**

**Hip surgery**

| I33 | Hip Replacement for Non-Trauma |
| --- | --- |
| I33A | Hip Replacement for Non-Trauma, Major Complexity |
| I33B | Hip Replacement for Non-Trauma, Minor Complexity |
| I03 | Hip Replacement for Trauma |
| I03A | Hip Replacement for Trauma, Major Complexity |
| I03B | Hip Replacement for Trauma, Minor Complexity |
| I31 | Revision of Hip Replacement |
| I31A | Revision of Hip Replacement, Major Complexity |
| I31B | Revision of Hip Replacement, Intermediate Complexity |
| I31C | Revision of Hip Replacement, Minor Complexity |

**Knee surgery**

| I04 | Knee Replacement |
| --- | --- |
| I04A | Knee Replacement, Major Complexity |
| I04B | Knee Replacement, Minor Complexity |
| I32 | Revision of Knee Replacement |
| I32A | Revision of Knee Replacement, Major Complexity |
| I32B | Revision of Knee Replacement, Minor Complexity |

**Appendix 2. List of pain medicines that we searched for.**

| Analgesics (N02B) |
| --- |
| Paracetamol |
| Anti-inflammatory and antirheumatic products, non-steroids (M01A) |
| Aspirin (acetylsalicylic acid) when doses >=150mg/day |
| Diclofenac |
| Ibuprofen |
| Naproxen |
| Piroxicam |
| Celecoxib |
| Indomethacin |
| Ketorolac |
| Ketoprofen |
| Parecoxib |
| Etoricoxib |
| Opioid anaesthetics (N01AH) and Opioids (N02A) |
| Oxycodone |
| Fentanyl |
| Buprenorphine |
| Tramadol |
| Morphine |
| Hydromorphone |
| Tapentadol |
| Codeine phosphate |
| Alfentanil |
| Sufentanil |
| Remifentanil |
| Fentanyl, combinations |
| Codeine and paracetamol |
| Oxycodone and naloxone |
| Muscle relaxants (M03) |
| Orphenadrine |
| Baclofen |
| Antiepileptics (N03A) |
| Gabapentin |
| Topiramate |
| Pregabalin |
| Carbamazepine |
| Antidepressants (N06A) |
| Amitriptyline |
| Nortriptyline |
| Duloxetine |
| Venlafaxine |
| Anaesthetics N01 |
| Ketamine |

**Appendix 3. Opioid Dose Equivalence Calculation Table**

**Source:** [**Australian and New Zealand College of Anaesthetists 2021**](https://www.anzca.edu.au/getattachment/6892fb13-47fc-446b-a7a2-11cdfe1c9902/PS01(PM)-(Appendix)-Opioid-Dose-Equivalence-Calculation-Table)

| OPIOID | Unit | Conversion factor | Proprietary names |
| --- | --- | --- | --- |
| ORAL (SWALLOWED) PREPARATIONS  Note: Modified release formulations are marked MR | | | |
| Morphine | mg/day | 1 | Anamorph, Kapanol (MR), MS Contin (MR), MS Mono (MR), Ordine, Sevredol |
| Oxycodone | mg/day | 1.5 | Endone, OxyContin (MR), OxyNorm, Targin (MR) |
| Hydromorphone | mg/day | 5 | Dilaudid, Jurnista (MR) |
| Codeine | mg/day | 0.13 | Aspalgin, Codalgin, Panadeine, Panadeine Forte, Mersyndol, Nurofen Plus, others |
| Dextropropoxyphene | mg/day | 1 | Di-Gesic, Doloxene |
| Tramadol | mg/day | 0.2 | Durotram-XR (MR) , Tramal, Tramadol SR (MR), Zydol, Zydol SR (MR), others |
| Tapentadol | mg/day | 0.3 | Palexia-SR (MR), Palexia-IR |
|  |  |  |  |
| SUBLINGUAL PREPARATIONS | | | |
| Buprenorphine | mg/day | 40 | Suboxone, Subutex, Temgesic |
|  |  |  |  |
| RECTAL PREPARATION  Note: Absorption from rectal administration is highly variable | | | |
| Oxycodone | mg/day | 1.5 | Proladone |
|  |  |  |  |
| TRANSDERMAL PREPARATIONS | | | |
| Buprenorphine | mcg/hr | 2 | Norspan |
| Fentanyl | mcg/hr | 3 | Denpax, Durogesic, Dutran, Fenpatch, Fentanyl Sandoz |
|  |  |  |  |
| PARENTERAL PREPARATIONS | | | |
| Morphine | mg/day | 3 | DBL morphine sulphate injection, DBL morphine tartrate injection |
| Oxycodone | mg/day | 3 | OxyNorm FI |
| Hydromorphone | mg/day | 15 | Dilaudid FI, Dilaudid-HP FI |
| Codeine | mg/day | 0.25 | Codeine phosphate injection USP |
| Pethidine | mg/day | 0.4 | Pethidine injection BP |
| Fentanyl | mg/day | 0.2 | DBL fentanyl injection, Sublimaze |
| Sulfentanil | mg/day | 2 | **-** |

**Appendix 4. Distribution of total number of prescriptions**

**Appendix 5. Number of prescriptions per pain medication class (n = 7696 orders).**

|  | Hip replacement  (n = 3556) | Knee replacement  (n = 4140) | Total  (n = 7696) |
| --- | --- | --- | --- |
| Opioids | 2386 (67.1) | 2831 (68.4) | 5217 (67.8) |
| Oxycodone | 907 (25.5) | 1073 (25.9) | 1980 (25.7) |
| Tapentadol | 610 (17.2) | 939 (22.7) | 1549 (20.1) |
| Morphine | 258 (7.3) | 189 (4.6) | 447 (5.8) |
| Buprenorphine | 166 (4.7) | 254 (6.1) | 420 (5.4) |
| Fentanyl | 157 (4.4) | 186 (4.5) | 343 (4.5) |
| Oxycodone-naloxone | 127 (3.6) | 83 (2) | 210 (2.7) |
| Tramadol | 117 (3.3) | 92 (2.2) | 209 (2.7) |
| Hydromorphone | 40 (1.1) | 15 (0.4) | 55 (0.7) |
| Codeine | 4 (0.1) | 0 | 4 (0.05) |
| Paracetamol | 791 (22.2) | 883 (21.3) | 1674 (21.7) |
| Paracetamol | 791 (22.2) | 883 (21.3) | 1674 (21.8) |
| Non-steroidal anti-inflammatories | 142 (4) | 163 (3.9) | 305 (4) |
| Aspirin (dose ≥ 150 mg/day) | 54 (1.5) | 79 (1.9) | 133 (1.7) |
| Celecoxib | 44 (1.2) | 52 (1.3) | 96 (1.2) |
| Ibuprofen | 13 (0.4) | 14 (0.3) | 27 (0.4) |
| Diclofenac | 11 (0.3) | 8 (0.2) | 19 (0.3) |
| Indomethacin | 6 (0.2) | 2 (0.05) | 8 (0.1) |
| Naproxen | 5 (0.1) | 3 (0.1) | 8 (0.1) |
| Parecoxib | 4 (0.1) | 3 (0.01) | 7 (0.1) |
| Ketoprofen | 4 (0.1) | 0 | 4 (0.05) |
| Ketorolac | 1 (0.03) | 1 (0.02) | 2 (0.03) |
| Piroxicam | 0 | 1 (0.02) | 1 (0.01) |
| Antiepileptics | 87 (2.5) | 112 (2.7) | 199 (2.6) |
| Pregabalin | 72 (2) | 93 (2.2) | 165 (2.1) |
| Gabapentin | 11 (0.3) | 11 (0.3) | 22 (0.3) |
| Carbamazepine | 4 (0.1) | 6 (0.1) | 10 (0.1) |
| Topiramate | 0 | 2 (0.05) | 2 (0.03) |
| Corticosteroids | 94 (2.6) | 67 (1.6) | 161 (2.1) |
| Dexamethasone | 44 (1.2) | 35 (0.8) | 79 (1) |
| Prednisolone | 36 (1) | 24 (0.6) | 60 (0.8) |
| Hydrocortisone | 13 (0.4) | 8 (0.2) | 21 (0.3) |
| Methylprednisolone | 1 (0.03) | 0 | 1 (0.01) |
| Antidepressants | 47 (1.3) | 73 (1.8) | 120 (1.6) |
| Amitriptyline | 30 (0.8) | 32 (0.8) | 62 (0.8) |
| Venlafaxine | 10 (0.3) | 21 (0.5) | 31 (0.4) |
| Duloxetine | 7 (0.2) | 20 (0.5) | 27 (0.4) |
| Anaesthetics | 7 (0.2) | 11 (0.3) | 18 (0.2) |
| Ketamine | 7 (0.2) | 11 (0.3) | 18 (0.2) |
| Muscle relaxants | 2 (0.1) | 0 | 2 (0.03) |
| Baclofen | 2 (0.06) | 0 | 2 (0.03) |

Values may not add up to 100% due to rounding

**Appendix 6. Frequency (%) of surgeries that had at least one prescription for each medication and drug class stratified by revision status (primary versus revision).**

|  | Hip replacement  (n =558) | | Knee replacement  (n = 724) | |  |
| --- | --- | --- | --- | --- | --- |
|  | Primary  (n = 499) | Revision  (n = 59) | Primary  (n = 672) | Revision  (n = 52) | |
| Opioids | 498 (99.8) | 59 (100) | 672 (100) | 52 (100) | |
| Oxycodone | 437 (87.6) | 51 (86.4) | 585 (87.1) | 46 (88.5) | |
| Tapentadol | 277 (55.5) | 45 (76.3) | 446 (66.4) | 33 (63.5) | |
| Fentanyl | 138 (27.7) | 14 (23.7) | 167 (24.9) | 15 (28.9) | |
| Morphine | 152 (30.5) | 14 (23.7) | 152 (22.6) | 15 (28.9) | |
| Buprenorphine | 100 (20) | 22 (37.3) | 195 (29) | 15 (28.9) | |
| Tramadol | 79 (15.8) | 4 (6.8) | 61 (9) | 7 (13.5) | |
| Oxycodone-naloxone | 62 (12.4) | 12 (20.3) | 45 (6.7) | 7 (13.5) | |
| Hydromorphone | 24 (4.8) | 1 (1.7) | 12 (1.8) | 1 (1.9) | |
| Codeine | 3 (0.6) | 1 (1.7) | 0 | 0 | |
| Paracetamol | 498 (99.8) | 58 (98.3) | 671 (99.9) | 52 (100) | |
| Paracetamol | 498 (99.8) | 58 (98.3) | 671 (99.9) | 52 (100) | |
| Non-steroidal anti-inflammatories | 92 (18.4) | 14 (23.7) | 126 (18.8) | 12 (23.1) | |
| Aspirin (dose ≥ 150 mg/day) | 31 (6.2) | 6 (10.2) | 64 (9.5) | 3 (5.8) | |
| Celecoxib | 34 (6.8) | 3 (5.1) | 43 (6.4) | 3 (5.8) | |
| Ibuprofen | 10 (2) | 2 (3.4) | 9 (1.3) | 5 (9.6) | |
| Diclofenac | 8 (1.6) | 1 (1.7) | 6 (0.9) | 1 (1.9) | |
| Indomethacin | 3 (0.6) | 2 (3.4) | 1 (0.2) | 1 (1.9) | |
| Parecoxib | 4 (0.8) | 0 | 3 (0.5) | 0 | |
| Naproxen | 3 (0.6) | 1 (1.7) | 2 (0.3) | 0 | |
| Ketoprofen | 1 (0.2) | 1 (1.7) | 0 | 0 | |
| Ketorolac | 1 (0.2) | 0 | 1 (0.2) | 0 | |
| Piroxicam | 0 | 0 | 1 (0.2) | 0 | |
| Antiepileptics | 52 (10.4) | 12 (20.3) | 77 (11.5) | 14 (26.9) | |
| Pregabalin | 43 (8.6) | 10 (16.9) | 68 (10.1) | 12 (23.1) | |
| Gabapentin | 6 (1.2) | 2 (3.4) | 6 (0.9) | 2 (3.9) | |
| Carbamazepine | 3 (0.6) | 0 | 5 (0.7) | 0 | |
| Topiramate | 0 | 0 | 2 (0.3) | 0 | |
| Corticosteroids | 59 (11.8) | 6 (10.7) | 47 (7) | 5 (9.6) | |
| Dexamethasone | 39 (7.8) | 0 | 29 (4.3) | 1 (1.9) | |
| Prednisolone | 19 (3.8) | 5 (8.5) | 17 (2.5) | 3 (5.8) | |
| Hydrocortisone | 4 (0.8) | 2 (3.4) | 5 (0.7) | 1 (1.9) | |
| Methylprednisolone | 1 (0.2) | 0 | 0 | 0 | |
| Antidepressants | 33 (6.6) | 7 (11.9) | 60 (8.9) | 5 (9.6) | |
| Amitriptyline | 21 (4.2) | 6 (10.7) | 29 (4.3) | 1 (1.9) | |
| Venlafaxine | 7 (1.4) | 1 (1.7) | 19 (2.8) | 1 (1.9) | |
| Duloxetine | 6 (1.2) | 1 (1.7) | 12 (1.8) | 3 (5.8) | |
| Anaesthetics | 4 (0.8) | 3 (5.1) | 7 (1) | 3 (5.8) | |
| Ketamine | 4 (0.8) | 3 (5.1) | 7 (1) | 3 (5.8) | |
| Muscle relaxants | 1 (0.2) | 1 (1.7) | 0 | 0 | |
| Baclofen | 1 (0.2) | 1 (1.7) | 0 | 0 | |

**Appendix 7. Sensitivity analysis considering that 25% (OMEDD 25%), 75% (OMEDD 75%), and 100% (OMED 100%) of PRN opioid orders were prescribed to patients.**

|  | OMEDD (25%) | OMEDD (75%) | OMEDD (100%) |
| --- | --- | --- | --- |
| Total sample | 30.7  (17.6 – 51.6 | 71.1  (40.9 – 103.1) | 90.8  (51.7 – 127.9) |
| Type of surgery |  |  |  |
| Hip replacement | 27.9 (16.8 – 54.3) | 69.3 (37.1 – 104.1) | 87.7  (47.5 – 127.5) |
| Knee replacement | 32  (18 – 49.4) | 73.1  (44.2 – 102.4) | 92.9  (55.8 – 128.9) |
| Elective surgery |  |  |  |
| Yes | 31.6  (18.2 – 53.4) | 73.1  (43.9 – 105.5) | 93.6  (55.1 – 131.6) |
| No | 20.3  (12.7 – 34) | 41  (26.4 – 64.8) | 50.5  (32.4 – 82) |
| Complexity |  |  |  |
| Major | 29.5  (16 – 50.6) | 63.1  (33.2 – 96.4) | 79.7  (41.7 – 119.4) |
| Minor | 30.9  (18 – 52) | 72.3  (42.6 – 104.6) | 92.9  (54.2 – 131.5) |
| Revision surgery |  |  |  |
| Yes | 29.8  (18.1 – 51.9) | 68.1  (40.1 – 95.1) | 85.6  (48 – 117.1) |
| No | 30.8  (17.6 – 51.6) | 71.7  (40.8 – 104) | 91.2  (51.7 – 130) |
| Pain medicines received |  |  |  |
| Opioids and paracetamol only | 26.1  (15.4 – 43.9) | 63.2  (35.2 – 93.6) | 79.9  (43.8 – 117.8) |
| Opioids, paracetamol and other pain medicines | 39.6  (23.2 – 62.2) | 81.9  (48.9 – 119) | 102.5  (62.5 – 144.9) |
